# Influence of vocal and aerodynamics aspects on the voice-related quality of life of older adults

**DOI:** 10.1590/1678-7757-2020-0052

**Published:** 2020-08-17

**Authors:** Larissa Thaís Donalonso SIQUEIRA, Kelly Cristina Alves SILVERIO, Giédre BERRETIN-FÉLIX, Kátia Flores GENARO, Ana Paula FUKUSHIRO, Alcione Ghedini BRASOLOTTO

**Affiliations:** 1 Departament of Speech-Language Pathology Universidade Estadual do Centro-Oeste IratiParaná Brasil Departament of Speech-Language Pathology of the Universidade Estadual do Centro-Oeste , UNICENTRO, Irati , Paraná , Brasil .; 2 Departament of Speech-Language Pathology Faculdade de Odontologia de Bauru Universidade de São Paulo BauruSão Paulo Brasil Departament of Speech-Language Pathology of the Faculdade de Odontologia de Bauru da Universidade de São Paulo , Bauru , São Paulo , Brasil .

**Keywords:** Older adults, Voice, Breath, Quality of Life

## Abstract

The pursuit for quality of life urged a better understanding of aspects involved in ageing to minimize its consequences. Although many studies investigated older adults’ voice, aspects affecting this population voice-related quality of life have not yet been explored.

**Objective:**

To investigate how aerodynamics and vocal aspects are associated with voice-related quality of life in older adults.

**Methodology:**

fifty-six older adults aged 60 years or above – 39 women and 17 men – were evaluated. The following procedures were performed: application of the Voice-Related Quality of Life (V-RQOL) protocol; vocal assessment, including auditory-perceptual and acoustic analysis, from which we obtained fundamental frequency (F _0_ ), standard deviation of fundamental frequency (SDF _0_ ), shimmer, amplitude perturbation quotient (APQ), jitter, pitch period perturbation quotient (PPQ), and harmonics to noise ratio (HNR); aerodynamic assessment using a spirometer; and maximum phonation time (MPT) for /a/, /s/, /z/ and number counting.

**Results:**

older adults tend to present high V-RQOL scores. Among women, roughness, APQ, and HNR parameters were negatively correlated with V-RQOL, whereas F _0_ was positively. We found no correlation between spirometry measurements and V-RQOL. MPT for /a/, /z/, and number counting was positively correlated with V-RQOL solely among men.

**Conclusion:**

Vocal roughness and acoustic parameters have a negative impact on the quality of life of older women. Respiratory aspects related to the available air support for speaking affected the most the voice-related quality of life of older men.

## Introduction

The process of aging, in general, demonstrates a large individual variation in the organic and functional decline ^1^ that may affect the quality of life of the elderly, a better understanding of the issues involved in this process is necessary in order to minimize its consequences.

The quality of life depends on the interpretation and subjective perception that each individual makes of the facts and events relating to the events and conditions of their life. ^2 , 3^ It is therefore necessary to develop strategies for knowing how the elderly perceive their own aging.

Much like the quality of life, the impact of a specific health problem is difficult to measure. It is known that the degree of dysphonia is not directly proportional to the impact this exerts on the life of the dysphonic individual. ^4^ Many researchers have been devoted to the development of instruments to measure dysphonia and its impact. Among them, there is the V-RQOL, Voice Related Quality of Life, validated in Brazil, ^5^ which evaluates the relationship of the voice with the quality of life. According Romak, et al. ^6^ (2014), this protocol is more sensitive than VHI-10, when the goal is to identify the impact of voice on quality of life in the elderly.

The aging of the voice occurs in a manner that is parallel to the other body functions. Throughout a person’s life, the voice undergoes decay processes that depend on the way of life, ^7^ and a sequence of physiological events related to vocal fold aging. ^8^ In the elderly, decreased mobility of the laryngeal joints may occur due to ossification of cartilage, accompanied by atrophy of the vocal and laryngeal muscle, changes in the vocal fold cover and onset of glottal closure. ^9 - 12^ As for the vocal characteristics in the elderly, there is unstable voice, increase of the duration of articulatory pauses and speech rate reduction in voice; more breathy voice and of weak intensity, reduction of maximum phonation times. ^13 - 16^ Men and women may present anatomical differences in aging, such as edema in the female vocal folds, reducing the arch and incomplete glottal closure ^9^ and generate differences in vocal quality, as well as reduction in fundamental frequency in women ^17^ and an increase of such in men. ^13^ Vocal changes may be some of the factors that lead the elderly population to seek health services, noting, therefore, that the vocal conditions affect the overall quality of life of the elderly. Authors verified by acoustic analysis that there is deterioration of vocal quality in relation to aging and concluded that vocal therapy can improve the quality of life of elderly patients, leading to better vocal performance and social communicative interaction. ^8^

In addition to the laryngeal and vocal modifications, there are changes in the respiratory tract during the aging process negatively impacting aerodynamic measurements, ^18^ as insufficiency in respiratory support, reduction of the subglottic airway pressure with decreased vocal intensity and the maximum phonation time of the elderly. According to authors, changes in pulmonary function associated with glottic incompetence may have an intense impact on the vocal quality of this population. ^19^ It is also noteworthy that there is a difference in the vital capacity measures between elderly men and women, as well as the maximum phonatory time, and these measures are higher for males. ^20^ As in the larynx, the changes that occur in the respiratory system may hinder the oral communication of the elderly with family members and other people, and may cause social isolation with impairment in the quality of life of this population.

Thus, even though there are many studies on vocal aging, understanding the impact of respiratory and vocal characteristics on the quality of life of the elderly is still not much explored. No studies correlating changes in breathing with the quality of life in order to investigate if these factors lead to a poorer quality of life in voice in different individuals were found. Specifically in the elderly population, only one study correlated the auditory perceptual voice data with the quality of life in women, ^14^ revealing that even with high scores on quality of life protocols, the worse the voice quality, the greater impairment in the quality of life. In this sense, the hypothesis of this study is that changes resulting from vocal and/or respiratory aspects in elderly people interfere with their quality of life, and that this may occur similarly or differently in men and women. Know the interference these aspects in the quality of life of the elderly will to a broader understanding of the vocal problems and thus, speech therapy interventions in this population may be more effective.

The aim of this study was to investigate the correlation of vocal and aerodynamics of vocal function aspects with the quality of life in the voice of elderly men and women.

## Methodology

### Sample

The Ethics Committee of University (050/2011) approved this study and all seniors signed the consent form.

Included in this study were the elderly from 60 years of age who contemplate the inclusion criteria: No history of neurological, oncological, endocrinological, psychiatric diseases, of lower airway, laryngeal surgeries, alcoholics and current smokers or have ceased to carry these habits for over twenty years.

Elderly were guests from seniors groups and Bauru community. Thus, 56 seniors participated, aged between 60 and 86 years (mean 67.94 years). Of these, 39 were women with a mean age of 67.97 years and 17 were men with a mean age of 67.88 years. All elderly participants had healthy aging.

## Procedures

### Voice-related quality of life

The elderly were instructed to fill out the protocol for the Voice-Related Quality Of Life (V-RQOL) ^5^ which assesses the impact of dysphonia on the quality of life. The choice of this instrument is due to the fact that it is a rapid application, simple for the elderly to respond and effective for the objective that it pursues. When the elderly had difficulties to understand any word of the questions of the V-RQOL protocol, the evaluator explained the meaning of the word so that he could respond appropriately. The questionnaire has 10 items covering the physical functionality and socio-emotional domain. Their range of responses contains “it is not a problem”, “it’s a small problem,” “it is a moderate / medium problem,” “it is a big problem,” “it is a very big problem.” These responses are graded from 1 to 5, respectively. The calculation was performed according to the responses, the result of which can vary from 0 to 100 percent in which the results of higher value indicate a better quality of life. ^5^

### Vocal evaluation

The vocal recordings were performed in an acoustically treated room where the elderly were instructed to perform the vowel /a/ in habitual pitch and loudness as well as a sample of spontaneous conversation. As for spontaneous speech, it was solicited the elderly answer the questions: “How was your day yesterday?” and “Tell me about your routine”. These speech samples are captured by the AKG C 444 PP model microphone, positioned at 45 degrees to the front of the mouth, four centimeters away from the labial commissure and recorded directly into a computer system and Audigy II Creative brand model sound card. The recordings were made by professional audio editing software - Sound Forge 10.0 (Sony, New York, EUA) in 44.100 Hz sample rate, channel Mono in 16Bit.

For the auditory perceptual analysis, the samples were submitted in blind form and randomized to three judges with experience in auditory perceptual voice analysis and it was repeated 20% of the sample for intra judgement. Perceptual vocal attributes evaluated for the vowel /a/ were: overall degree of vocal deviation, roughness, breathiness, strain and instability; for spontaneous speech, the overall degree of vocal deviation was analyzed. The evaluation was performed by means of a visual analog scale of 100 millimeters, ^17^ in which the reviewer stated, for each parameter, the degree of deviation, using a vertical line on the scale, and the extreme left means no voice alteration for the evaluated parameter and its extreme right means the presence of the change in maximum degree. The average score given by the judges for each attribute was considered as result of the degree of vocal deviation.

## Acoustic voice analysis

The acoustic voice analysis was performed using the Sound Forge 10.0 software, and the recordings of the vowel /a/ sustained, having its beginning and its end excluded in order to eliminate the main stretches of vocal instability. Through the Multi Dimension Voice Program (MDVP) computerized program, Model 5105, from KayPENTAX ( *Kay Elemetrics* , Linkoln Park, NJ), the following acoustic parameters were extracted: Fundamental frequency (F _0_ ) and Standard Deviation of F _0_ (SD F _0_ ), Pitch Perturbation Quotient (PPQ), Amplitude Perturbation Quotient (APQ) and noise to harmonic ratio (NHR).

### Spirometry

The Pony FX 12 (COSMED, Rome, Italy) liter spirometer was used for the spirometric evaluations. A nozzle with its filter coupled to a spirometer and the plastic tube was placed in the vestibule of the mouth of the elderly, being asked to breathe normally until they got used to the system. The elderly remained comfortably seated in a chair and then the elderly performed maximal inspiration, pause for a few seconds and a maximum expiration in the form of forced breath. The measurement of vital capacity (VC) was performed without occluding the nostrils (since this measure represents the normal breathing situation of the elderly), by asking the elderly to expire all the air in the mouth of the tube unit. This procedure was repeated three times, the average calculated in liters. ^18^ For quantitative evaluation of the pneumophonoarticulatory coordination the same equipment was used, graphically recording the phonation volume (PV), measured in liters, and the average flow phonation (AFP), calculated in liters per second, obtained from prolonged vowel /a/ in the mouthpiece of the spirometer. The elderly were instructed to perform a maximal inspiration followed by prolonged vowel /a/, this procedure was performed three times and averaged. The simple phonic quotient (SPQ) obtained by evaluating the ratio between the VC and the MPT of the vowel / a / was also calculated.

## Maximum phonation time

The maximum phonation time (MPT) was obtained by requesting a prolonged emission in one expiration following a deep breath, of the vowel /a/, the fricatives /s/ and /z/ and counting numbers, with each emission performed three times, and the average values were calculated. To measure the MPT, the same voice recording equipment was used.

## Statistical analysis

The correlation of all data assessed with the V-RQOL protocol was performed using the Spearman correlation. The significance level of 5% was adopted for all statistical analyzes.

The inter and intra judgement reliability was performed using the Intraclass correlation coefficient (ICC) in that the interpretation of the values ^21^ is poor when it is less than 0.4, satisfactory between 0.4 and 0.74 and excellent when most equal to 0.75. The results for the inter judgement agreement were excellent for all parameters, ranging from 0.744 to 0.830. The result was satisfactory with a value of 0.704 for just the “tension” parameter. Regarding the evaluation of the intra judgement reliability, all the results were excellent, ranging from 0.752 to 0.896.

We adopted the following interpretation of correlation coefficients for the negative and positive correlations: the correlation coefficient from 0.70 to 1.0 as an indication of a strong correlation; between 0.40 and 0.60, moderate correlation; between 0.10 and 0.3, weak correlation. ^22^

## Results

Table 1-3 [Table t2] [Table t3] shows the mean values of all evaluated aspects of the elderly in general. Emphasized that it is possible to verify that some older people had low scores on the protocol, which is compatible with dysphonia. ^23^

**Table 1:** Scores V-RQOL Protocol of men and women elderly

Parameters	Mean		Mean		Mean	
	Total Elderly		Women		Men	
	Mean (SD)	Minimum-maximum	Mean (SD)	Minimum-maximum	Mean (SD)	Minimum-maximum
V-RQOL Social-emotional (%)	97.66 (±8.47)	50-100	97.92(±8.28)	50-100	97.06(±9.14)	62.5-100
V-RQOL Physical (%)	95.76 (±7.17)	66.67-100	96.58(±5.39)	79.17-100	93.88(±10.11)	66.67-100
V-RQOL Total (%)	96.52 (±6.77)	67.5-100	97.12(±5.51)	70-100	95.15(±9.08)	67.5-100

**Table 2 t2:** Values of parameters of the perceptual analyze and acoustic parameters of men and women elderly

Parameters	Total Elderly		Women		Men	
	Mean (SD)	Minimum-maximum	Mean (SD)	Minimum-maximum	Mean (SD)	Minimum-maximum
Global degree of vocal quality - /a/ (mm)	41.30(±12.13)	17.4-64.4	40.66(±12.95)	17.4-64.4	42.73(±10.27)	31.4-62
roughness - /a/ (mm)	23.90(±14.03)	0-60.33	20.32(±12.93)	0-60.33	31.92(±13.37)	12.67-60
breathiness - /a/ (mm)	22.10(±11.18)	6-48.67	20.82(±11.01)	6-48.67	24.84(±11.4)	9.0-48
strain - /a/ (mm)	9.28(±8.66)	0-29.67	10.49(±9.55)	0-29.67	6.57(±5.58)	0-18.33
Instability - /a/ (mm)	29.30(±8.66)	9.67-62.67	31.44(±13.81)	12-62.67	25.78(±11.66)	9.67-56.33
Global degree of vocal quality – spontaneous speech (mm)	34.1(±11.95)	12.33-60	33.28(±13.32)	12.33-60	37.34(±7.58)	23.33-51.33
fundamental frequency (Hz)	177.48(±44.89)	92.64-279.68	199.05(±32.06)	117.26-279.68	128.00(±27.39)	92.64-181.26
SD F0 (Hz)	4.02(±3.57)	1.08-23.14	4.31(±4)	1.37-23.14	3.35(±2.29)	1.08-9.77
PPQ (%)	0.82(±0.63)	0.16-3.5	0.73(±0.5)	0.16-2.18	1.02(±0.85)	0.19-3.5
APQ (%)	3.13(±2.66)	0.91-19.55	2.53(±1.35)	0.91-7.56	4.46(±4.11)	1.76-19.55
NHR (dB)	0.15(±0.06)	0.09-0.46	0.14(±0.04)	0.1-0.27	0.17(±0.08)	0.09-0.46

**Table 3 t3:** Values of respiratory parameters of men and women elderly

Parameters	Mean		Mean		Mean	
	Total Elderly		Women		Men	
	Mean (SD)	Minimum-maximum	Mean (SD)	Minimum-maximum	Mean (SD)	Minimum-maximum
Vital Capacity (litres)	2.46(±0.76)	1.24-4.21	2.14(±0.49)	1.24-3,44	3.20(±0.77)	1.47-4.21
Phonation volume (litres)	2.80(±1.07)	1.08-5.23	2.42(±0.78)	1.09-4.56	3.72(±1.14)	1.08-5.23
Average Flow Phonation (litres/seconds)	0.24(±0.13)	0-0.68	0.21(±0.09)	0.08-0.47	0.32(±0.18)	0-0.68
Simple Phonic Quotient (litres/seconds)	0.22(±0.11)	0.06-0.61	0.19(±0.07)	0.06-0.33	0.29(±0.14)	0.1-0.61
MPT /a/ (seconds)	14.03(±6.71)	4-36.5	12.67(±5.29)	5-25.2	17.15(±8.58)	4-36.5
MPT /s/ (seconds)	12.37(±6.69)	4.3-40	10.55(±3.86)	4.3-20.27	16.54(±9.58)	4.3-40
MPT /z/ (seconds)	12.82(±5.70)	4.3-28.6	11.85(±4.58)	4.6-27.7	15.04(±7.39)	4.3-28.6
MPT numbers (seconds)	14.89(±5.90)	4.3-32.30	14.06(±4.85)	7.70-29.2	16.81(±7.63)	4.3-32.3

It is noteworthy that the analysis of the evaluations for the total group proves that there are no differences between the groups of men and women in the analyzed parameters.

**Figure 1:**
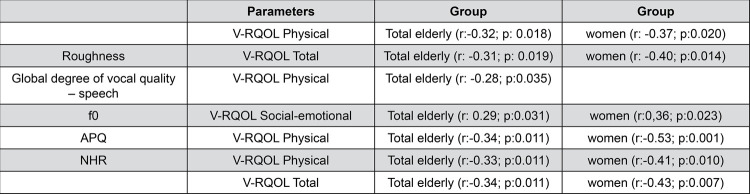
Significant correlations of the parameters of the perceptual analyze and acoustic parameters with V-RQOL

**Figure 2:**
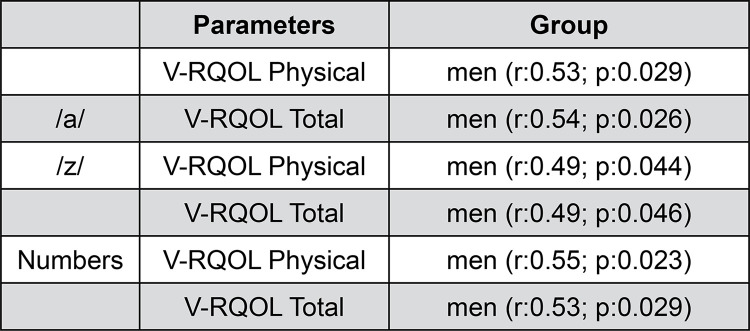
Significant correlations of MPT with V-RQOL Protocol

Figures 1 and 2 show statistically significant correlations of the aspects evaluated with the V-RQOL Protocol. No correlation of the spirometry data with the V-RQOL protocol was observed.

The correlations between the data evaluated with the V-RQOL protocol proved to be of moderate strength for the groups of elderly men and women, except for roughness vs physical V-RQOL and F _0_ vs socio-emotional V-RQOL for women (weak correlation). The strength of the correlations with the total group of seniors was weak.

## Discussion

Regarding the voice-related quality of life, the elderly had average scores in the V-RQOL protocol that indicate a good quality of life in voice in general, but it is observed that some elderly had low scores with greater impact in the quality of life. This finding may be indicative that some elderly had vocal disorders ^23^ ( Table 1 ).

Others studies have demonstrated V-RQOL scores in the elderly with scores indicating good quality of life, on average, ^24 - 30^ revealing that the vocal problems do not affect the quality of life in the perception of the elderly, since only the scores near 65.9% demonstrated significant vocal problems. ^5^ A high score on the V-RQOL protocol can be explained since this population perceives little vocal aging and most strategies to rejuvenate the voice are unknown, ^24^ or even for those that realize vocal changes, do not report complaints. One study revealed that there are no differences in the V-RQOL scores between elderly men and women, as well as in the different categories of young-old, middle-old and old-old. ^29^

In addition, it is emphasized that the elderly people may have little insight about voice changes due to aging because they believe that such changes are part of this stage of life, which contributes to not have complaints. Some authors have shown that the normal gradual process of vocal aging brings clear consequences in daily life that should be taken into account in clinical practice and in the communication of the social life of the elderly since voice disorders in this population are frequent and bring a negative impact in the quality of life and may also interfere with the physical functioning with social and emotional limitations. ^30 - 32^ Is worth emphasizing that this study did not aim to create a specific protocol for the elderly. Thus, it was not possible to change the V-RQOL protocol in full and for this reason, the issue related to work was kept in the analysis. It is emphasized that a scale of quality of life in voice, called Aging Voice Index (AVI), for elderly people, was developed, ^33^ but was not validated for Brazilian Portuguese, which may be considered a limitation of this study. In this way the V-RQOL protocol was used because it is easy to apply to this population.

Most of the present study were not active older workers, which may have contributed to better quality of life for elderly people with high scores in the physical and total domains. The Correlation analysis between the values of deviation from the auditory perceptual parameters with the values of the V-RQOL protocol, in the total elderly group, demonstrated that the overall grade and roughness were the parameters that were correlated with the physical domain and roughness correlated with the total score of the V-RQOL ( Figure 1 ). This means that the elderly with rougher voices and with a greater overall degree of deviation demonstrated feeling more impact of the vocal conditions in their lives, especially regarding the physical aspects. Authors correlated the parameters of the GRBASI scale with the domains of the V-RQOL protocol, and they found a negative correlation between the physical domain and the total score of V-RQOL degree with general parameter degrees, roughness, breathiness and instability. ^14^ In other words, the higher the vocal deviation, the worse the quality of life of the elderly. The data found in this study corroborated those from Gama, et al. ^14^ (2009).

The overall degree of vocal deviation of spontaneous conversation was correlated with the physical domain for all of the elderly. The overall grade may reflect a summation of several vocal aspects. For some authors, ^14^ certain alterations associated with aging, such as changes in voice quality, vocal instability and effort to speak may cause limitations in performing daily tasks. Other authors ^14 , 34^ reported that the higher the degree of dysphonia, the lower the quality of life related to voice.

The analysis in the subgroups of men and women indicated a correlation between roughness and the physical domain and the total V-RQOL score only for the women (Table 4). The same was found in another study, ^14^ in which the parameters of the GRBASI scale correlated only with the physical domain and the total score of the V-RQOL protocol.

Regarding the acoustic parameters (Table 4), in the correlation analysis of the F _0_ with the V-RQOL protocol, there was a positive correlation with the socio-emotional domain for the subgroup of women and for the total group of elderly, revealing that the more acute the voice, the better the quality of life of the women elderly. This interferes with the quality of life of elderly women and may be due to the fact that a lower pitched voice during aging process, when compared to the voice of their entire young and adult life, usually associated with higher roughness. This can occur because there is a possible presence of edema of the vocal folds, caused after menopause, and therefore, it can affect the quality of life and communication regarding the socio-emotional aspect. It is noteworthy that the correlations of this parameter with the V-RQOL protocol were found only for women and for the total group of elderly, not reflecting impairment in the quality of life of men. In the correlation analysis with the V-RQOL protocol, it was found that the APQ and NHR parameters were negatively correlated with the physical domain and the total score of the V-RQOL for the total elderly group and for the subgroup of women, revealing that, the greater the perturbations of amplitude of voice signal and noise, the worse the quality of life. This can also be explained by the fact that the larger the necessity to increase the voice intensity, the greater the noise in the speech signal and the increased instability. Vocal alterations can produce changes in the acoustic parameters with consequent loss of the quality of life for the elderly.

As the correlation analysis of spirometry with the V-RQOL Protocol, there was no correlation with the spirometric data with the domains and the total score of the V-RQOL, evidencing that respiratory issues do not impact the quality of life of the elderly people which can be reinforced by the result that there was no correlation of MPT /s/ with V-RQOL, since this phoneme is not produced by vibration of the vocal folds. Several researches related to spirometry and the respiratory system were consulted for this study in different populations, because little is known about this aspect and the use of measures from this test, especially in the healthy population over 60 years of age. Spirometry is an appropriate and accessible examination for the elderly, for the control and quantification of pulmonary diseases, in addition to compare and monitor examinations performed in previous years and in other centers of reference since it follows an international standardization. ^35^ Furthermore, spirometry is an instrumental examination that assists in the completion of data related to speech functions, by measuring the VC (maximum volume of air that can be expelled by stress at maximum inspiration) and quantitative evaluation of pneumophonoarticulatory coordination. As the acoustic parameters of the voice, the aerodynamic measures are objective measures to be used continuously to complement the vocal diagnosis, one of the consecrated tools for assessment of vocal behavior. ^35^ In the present study, elderly people who had problems with the lower airways such as asthma were excluded, because it is observed that individuals with such problems have reduced spirometric measures, as well as the MPT, besides presenting stiffness and body tension and vocal variations, which could influence the results. ^36^

Pulmonary function is influenced by aging, since there is a difference in the breathing pattern of young adults and the elderly. ^37^ Advancing age is associated with a reduction in inspiratory and expiratory muscle strength, whereas in women there is lower maximal expiratory pressure. ^38^ As for other authors, ^39^ the respiratory system is not impacted with the aging process, no difference in the respiratory pattern of young adults and elderly. However, authors concluded that pathological presbyphonia is influenced by respiratory capacity and gender, and respiratory capacity may be a point to be worked on in presbyphonia. ^40^

Despite the correlation analysis does not demonstrate cause-effect measures, we can observe in this study that the MPT may cause a reduced negative impact on their quality of life of the elderly men, which was not seen in the group of women.

Thus, it can be observed in the present study, that the spirometric data do not correlate with the quality of life of the elderly, both men and women (Figures 1 and 2). It is observed that when this population needs to coordinate speech with breath, it present difficulties, causing a worsening in their quality of life in voice, and affecting only the men ( Figure 2 ). Ie, the higher the value the MPT values for men, the better their quality of life in voice in the physical and the total dominions. Joshi ^20^ (2020) observed that the elderly expressed difficulties with aspects related to vocal projection, breathing support and pneumophonic coordination, however, these subjects presented a good quality of life in voice. Another study ^41^ analyzed that the elderly had greater difficulty breathing and talking when they needed to talk in noisy environments, producing shorter speeches and pulmonary adjustments to speak with greater intensity. Thus, this author concludes that vocal intensity and the duration of the speech have an influence on the respiratory dynamics during speech in the aging process. Authors also report that the association of changes in pulmonary function with glottic incompetence in the elderly can significantly affect voice quality, ^19^ which damages your quality of life.

Limitations of this study can be attributed to limited participation of healthy elderly men. Also, because it is a non-specific protocol for this population, the scores of the protocol presented high, with possible masking other aspects that may influence the quality of life in voice of the elderly population.

Based on such, these results show us the direction to follow in speech therapy, since it shows the specific needs of the elderly, being different for each sex. This fact can be confirmed by the strength of the statistical correlations between variables. The weak correlation was found in the total group of elderly, while subgroups of elderly men and women had moderate strength correlations, showing that each sex has its own peculiarities and differences in the impact of respiratory and vocal changes, even in a group of elderly perception of vocal little interference in their lives. Perhaps future studies with only elderly people with vocal complaints show stronger correlations than those found in this study, what can be considered as a limitation for the present study. Future studies comparing elderly people who are vocally healthy and elderly people with presbyphonia may demonstrate another type of influence of these aspects on quality of life. In addition, some assessments such as aerodynamics, should be better exploited to fill gaps, since there is limited information on such measures, ^42^ especially in the elderly, besides conducting multidimensional assessments in order to gain holistic understanding of the vocal problem. ^20^

Often in speech therapy, the therapy is focused from the alterations found in the larynx examinations and changes recognized in the auditory perceptual evaluations. However, only those evaluations are not enough for a good therapeutic outcome, and it is necessary to understand what actually affects the lives of the elderly, ie, that which hinders their communication. It is noteworthy that there are still not protocols for the quality of life in specific voice for this population, which could punctually help what impacts the lives of the elderly.

## Conclusion

The vocal aspects of roughness and acoustic parameters had a negative impact on the quality of life among elderly women, which did not occur among the men. The aerodynamics of vocal function aspects related to air support available to speak negatively influenced the quality of life of elderly men.
